# Ag-Mg antisite defect induced high thermoelectric performance of α-MgAgSb

**DOI:** 10.1038/s41598-017-02808-8

**Published:** 2017-05-31

**Authors:** Zhenzhen Feng, Jihua Zhang, Yuli Yan, Guangbiao Zhang, Chao Wang, Chengxiao Peng, Fengzhu Ren, Yuanxu Wang, Zhenxiang Cheng

**Affiliations:** 1 Institute for Computational Materials Science, School of Physics and Electronics, Henan University, Kaifeng, 475004 China; 2 Guizhou Provincial Key Laboratory of Computational Nano-Material Science, Guizhou Education University, 115 Gaoxin Road, Guiyang, 550018 China; 3 Institute for Superconducting and Electronic Materials, Innovation Campus, University of Wollongong, Squires Way, North Wollongong, NSW 2500 Australia

## Abstract

Engineering atomic-scale native point defects has become an attractive strategy to improve the performance of thermoelectric materials. Here, we theoretically predict that Ag-Mg antisite defects as shallow acceptors can be more stable than other intrinsic defects under Mg-poor‒Ag/Sb-rich conditions. Under more Mg-rich conditions, Ag vacancy dominates the intrinsic defects. The *p*-type conduction behavior of experimentally synthesized α-MgAgSb mainly comes from Ag vacancies and Ag antisites (Ag on Mg sites), which act as shallow acceptors. Ag-Mg antisite defects significantly increase the thermoelectric performance of α-MgAgSb by increasing the number of band valleys near the Fermi level. For Li-doped α-MgAgSb, under more Mg-rich conditions, Li will substitute on Ag sites rather than on Mg sites and may achieve high thermoelectric performance. A secondary valence band is revealed in α-MgAgSb with 14 conducting carrier pockets.

## Introduction

Thermoelectric materials can perform direct conversion between electrical and thermal energy. Thermoelectric performance is quantified by the figure of merit, ${ZT}\,=\,{S}^{2}{\rm{\sigma }}T/{\rm{\kappa }}$, where *S* is the Seebeck coefficient, *σ* is the electrical conductivity, *T* is the absolute temperature, and *κ* is the total thermal conductivity, which consists of both electronic (*κ*_*e*_) and lattice (*κ*_*l*_) components^1–3^. A high *ZT* value indicates good thermoelectric properties, and one therefore should try to increase the power factor (${S}^{2}\sigma$) and decrease the thermal conductivity (*κ* = *κ*_*e*_+*κ*_*l*_). A large power factor can be achieved by (a) increasing the density of states near the Fermi level (by forming localized resonant states^4, 5^ or increasing band degeneracy^6–16^), and (b) by increasing the energy dependence of the carrier mobility using energy filtering^17, 18^. Meanwhile, forming a solid solution^19–21^ and creating strong lattice anharmonicity^22–28^ can achieve low lattice thermal conductivity. A recent study has proposed that engineering atomic-scale native point defects can simultaneously optimize the thermal and electrical performances of thermoelectric materials^29, 30^, which is becoming an attractive strategy to improve *ZT* values. Native point defects play important roles in conduction in semiconductors, and they can change the band structure^29^.

The α phase of MgAgSb^31^ shows superior thermoelectric properties in the low temperature range^31–44^. Great efforts have been devoted to understanding and enhancing the unique thermoelectric properties of α-MgAgSb. The carrier concentration of α-MgAgSb-based materials is relatively low at room temperature, which leads to its high electrical resistivity. To overcome this limitation, extrinsic doping, including Na doping^35^, Cu doping^36^. In doping^38^, and changing the Sb content^41^ have been used to increase the carrier concentration of α-MgAg_0.97_Sb_0.99_ or α-MgAgSb, although the electrical resistivity (1‒4.5 × 10^−5^ Ω·m) is still larger than those of good thermoelectric materials, such as CoSb_3_ (0.3‒1 × 10^−5^ Ω·m)^45^ and Bi_2_Te_3_ (1‒1.5 × 10^−5^ Ω·m)^46^. Liu *et al*. used Li doping to increase the carrier concentration of MgAg_0.97_Sb_0.99_, and a high average *ZT* ^39^ of 1.1 from 300 K to 548 K was achieved.

Intrinsic defects represent another effective way to tune the carrier concentration to enhance the thermoelectric performance. Moreover, extrinsic point defects strongly influence the native point defects. Recently, Liu *et al*. reported that Ag vacancy could increase the *ZT* for α-MgAgSb^30^. Moreover, the Ag vacancy concentration can be tuned by the hot pressing temperature, which they denoted as the recovery effect. Therefore, it is necessary to explore the conditions for forming intrinsic defects and their influence on the electronic structure.

In this work, the chemical potentials and defect formation energies of native point defects and Li doping in α-MgAgSb at all possible charge states are studied by using density functional theory. We found that the defect formation energies strongly depend on the chemical potentials. Ag vacancies and Ag-Mg antisites (Ag on Mg sites) are the dominant defects that act as shallow acceptors, which determine the *p*-type conduction. Moreover, the Ag_Mg_ point defect in α-MgAgSb may have higher *ZT* than the Ag vacancy. For Li-doped α-MgAgSb, the doping formation energies strongly depend on the chemical potentials. Under more Mg-rich conditions, Li will substitute on Ag sites (Li_Ag_) rather than on Mg sites (Li_Mg_), and a larger *ZT* can be achieved by Li_Ag_ doping than by Li_Mg_ doping. By reasonably controlling the chemical potential, both the antisite defect Ag_Mg_ and Li substitution on Ag sites of α-MgAgSb can be obtained, and the products may be promising thermoelectric materials for low temperature power generation.

## Results and Discussion

### Chemical potentials and formation energies of native point defects

Engineering intrinsic defects may be an effective way to improve the thermoelectric performance of α-MgAgSb. Due to the complex phase transitions and the appearance of secondary phases, previous experimental works have shown that it is difficult to synthesize pure phase α-MgAgSb. Different types of native point defects may easily appear in α-MgAgSb. Thus, it is necessary to first explore the conditions for forming intrinsic point defects in α-MgAgSb.

The defect formation energy $({\rm{\Delta }}{H}_{f})$ is defined as

1 $${\rm{\Delta }}{H}_{f}={E}_{defect}^{q}-{E}_{perfect}+\,\sum _{i}{n}_{i}{\mu }_{i}+q({E}_{F}+{E}_{V}+\,{\rm{\Delta }}V),$$

where ${E}_{defect}^{q}$ is the total energy of the supercell with the incorporated defect, ${E}_{perfect}$ is the total energy of the supercell without the incorporated defect, ${n}_{i}$ is the number of atoms being removed or added, and ${\mu }_{i}$ is the corresponding chemical potential, ${E}_{F}$ is the Fermi energy, ${E}_{V}$ is the energy with respect to the valence band maximum (VBM), and Δ*V* is the average difference between the local potentials far from the defect in the defective supercell and the corresponding ones in the perfect supercell^47^.

We calculated the accessible range of chemical potentials for the equilibrium growth conditions of α-MgAgSb. Under equilibrium conditions for the crystal growth, the steady production of the host material, α-MgAgSb, should satisfy the following equations:

2 $${\mu }_{{MgAgSb}}=\,{\mu }_{{Mg}}+{\mu }_{{Ag}}+{\mu }_{{Sb}},$$

3 $${E}_{{MgAgSb}}={E}_{{Mg}}+{E}_{{Ag}}+{E}_{{Sb}}+{\rm{\Delta }}{H}_{f}({MgAgSb}),$$

4 $${\rm{\Delta }}{\mu }_{{Mg}}+{\rm{\Delta }}{\mu }_{{Ag}}+{\rm{\Delta }}{\mu }_{{Sb}}={\rm{\Delta }}{H}_{f}({MgAgSb}),$$

where ${\mu }_{{Mg}}$, ${\mu }_{{Ag}}$, and ${\mu }_{{Sb}}$ are the chemical potentials of Mg, Ag, and Sb, respectively, and ${\rm{\Delta }}{H}_{f}({MgAgSb})$ is the formation energy for α-MgAgSb. To avoid the precipitation of source elements, ${\rm{\Delta }}{\mu }_{{Mg}}$, ${\rm{\Delta }}{\mu }_{{Ag}}$, and ${\rm{\Delta }}{\mu }_{{Sb}}$ should satisfy:

5 $${\rm{\Delta }}{\mu }_{{Mg}} < {\rm{0}},{\rm{\Delta }}{\mu }_{{Ag}} < {\rm{0}},{\rm{\Delta }}{\mu }_{{Sb}} < 0.$$

To maintain the stability of MgAgSb during growth and avoid any competing phases (such as MgAg, Mg_3_Sb_2_, and Ag_3_Sb), the chemical potential ${\rm{s}}\,{\rm{\Delta }}{\mu }_{{Mg}}$, ${\rm{\Delta }}{\mu }_{{Ag}}$, and ${\rm{\Delta }}{\mu }_{{Sb}}$ must satisfy the following limits:

6 $${\rm{\Delta }}{\mu }_{{Mg}}+{\rm{\Delta }}{\mu }_{{Ag}} < {\rm{\Delta }}{H}_{f}({MgAg}),$$

7 $$3{\rm{\Delta }}{\mu }_{Mg}+2{\rm{\Delta }}{\mu }_{Sb} < {{\rm{\Delta }}H}_{{f}}(M{g}_{3}S{b}_{2}),$$

8 $$3{\rm{\Delta }}{\mu }_{{Ag}}+{\rm{\Delta }}{\mu }_{{Sb}} < {\rm{\Delta }}{H}_{f}({A}{{g}}_{3}{Sb}).$$

All calculated heats of formation of ternary and binary compounds in this work are given per formula unit.

**Figure 1:**
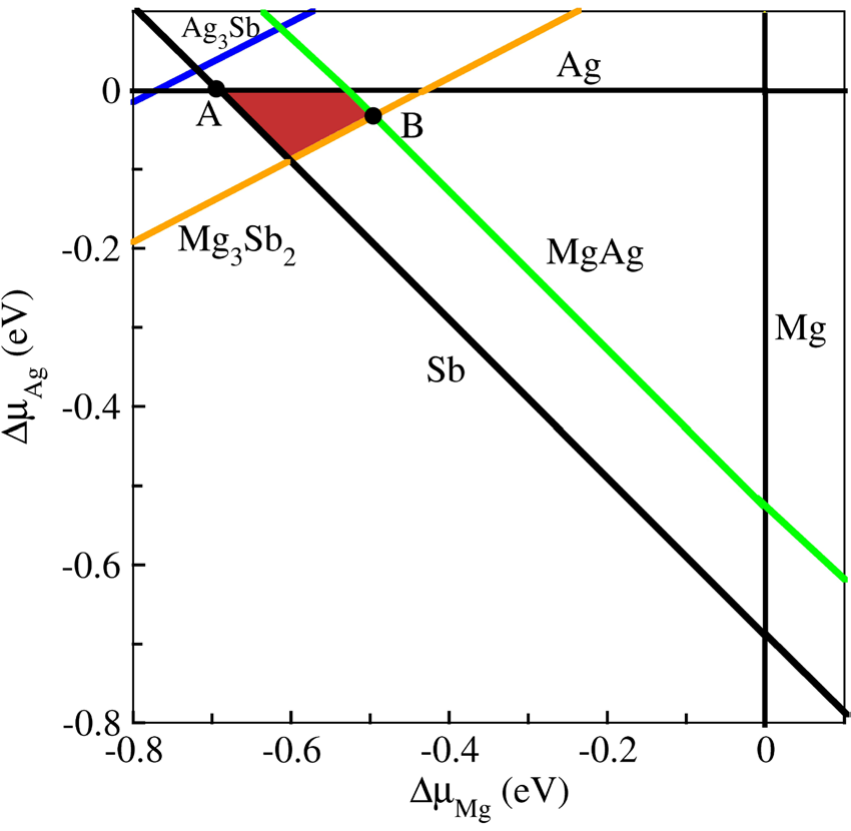
Accessible range of chemical potentials (shaded region) for equilibrium growth conditions of α-MgAgSb. The specific points A and B were chosen for the representative chemical potentials to be used for the following doping formation energy calculations.

Equations (4)–(8) can be projected onto the two-dimensional plane with two independent variables, ${\rm{\Delta }}{\mu }_{{Mg}}$ and ${\rm{\Delta }}{\mu }_{{Ag}}$, as shown in Fig. 1. The shaded region represents the area for the equilibrium growth conditions of α-MgAgSb. Figure 1 asserts that α-MgAgSb is only thermodynamically stable within a narrow Mg-Ag compositional range. The thermodynamically stable ranges of chemical potentials for the elements in α-MgAgSb are obtained by excluding the regions of chemical potentials in which competing phases are thermodynamically stable. Here, we present the calculated values at two representative chemical potential points labeled as A (−0.69 eV, 0, 0) and B (−0.469 eV, −0.032 eV, −0.162) in Fig. 1 for ${\rm{\Delta }}{\mu }_{{Mg}}$, ${\rm{\Delta }}{\mu }_{{Ag}}$, and ${\rm{\Delta }}{\mu }_{{Sb}}$, respectively.

**Figure 2:**
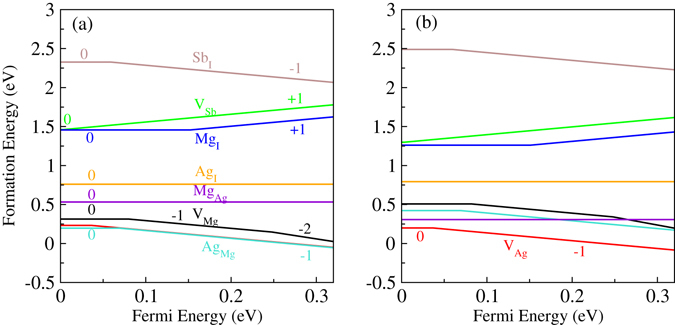
The calculated formation energies of native point defects in α-MgAgSb as a function of the Fermi level, with chemical potentials at (**a**) point A and (**b**) point B.

**Figure 3:**
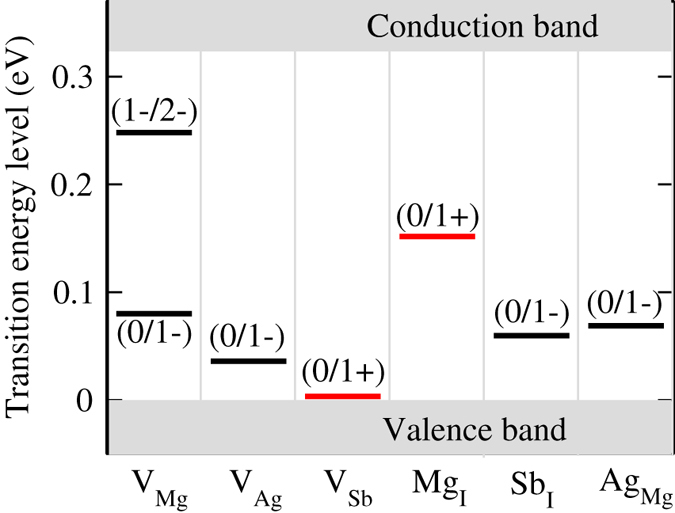
The calculated transition energy levels of various intrinsic defects in α-MgAgSb. The black bars show the acceptor levels, and the red bars show the donor levels.

To predict the conductivity type of MgAgSb with intrinsic defects, we calculated the Fermi level pinning positions. Figure 2(a) and (b) shows the calculated formation energies of native point defects as a function of the Fermi levels at chemical potential points A and B, respectively. The calculated transition energies for these defects are shown in Fig. 3. From the single-particle energy point of view, V_Mg_, V_Ag_, Sb_I_, and Ag_Mg_ should be acceptor-like defects, whereas V_Sb_, Mg_I_, Ag_I_, and Mg_Ag_ should be donor-like defects. Under Mg-poor‒Ag/Sb-rich conditions (point A in Fig. 1), the formation energy of the Ag_Mg_ antisite defect is very low, meaning that it is the dominant acceptor, and *p*-type conductivity can be realized by forming Ag_Mg_ antisite defects. The Ag_Mg_ antisite defect is thermodynamically stable. This suggests that Ag_Mg_ may stably exist in an Mg poor environment. Under more Mg-rich conditions (point B in Fig. 1), the V_Ag_ defect has the lowest formation energy, indicating that it is now the dominant type of acceptor, which is consistent with the results reported by Liu *et al*.^30^. Thus, our calculation results for the formation energy can explain why α-MgAgSb often exhibits *p*-type conductivity. From Fig. 3, it is seen that the transition energies of V_Ag_ and Ag_Mg_ are 0.036 eV and 0.068 eV above the VBM, respectively, indicating that V_Ag_ and Ag_Mg_ are shallow acceptors. On the other hand, all the defects that create deep levels, such as Mg_I_ and V_Mg_, have higher formation energies. Thus, the formation energies of the native point defects strongly depend on the chemical potentials, and Ag_Mg_ antisites and Ag vacancies are the dominant acceptor defects in α-MgAgSb. The calculated formation energy using chemical potentials is close to the real preparation environment. Under the different circumstances, we can compare the types of doping with which conditions are easier or more difficult to achieve, which can explain the experimental phenomena and provide a reference for controlling the defect type.

### Effects of native defects on electronic structure

**Figure 4:**
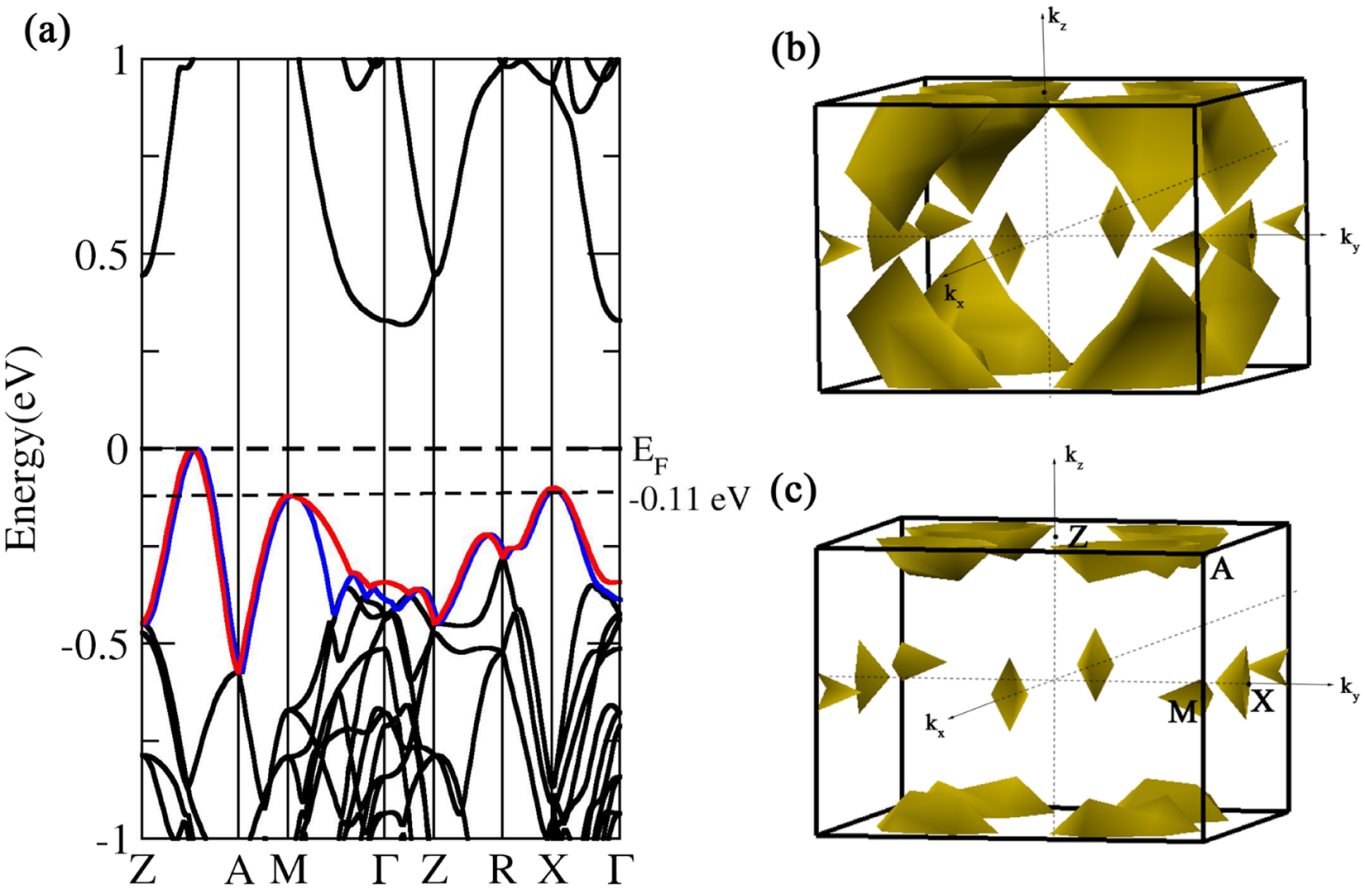
(**a**) Calculated band structure of α-MgAgSb. The top of the valence band is set to zero. The dashed lines denote the Fermi level. The special *k* points Z, A, M, Γ, R, and X are located at the points (0, 0, 0.5), (0.5, 0.5, 0.5), (0.5, 0.5, 0), (0, 0, 0), (0, 0.5, 0.5), and (0, 0.5, 0), respectively. (**b**) Fermi surface calculated for a Fermi level at −0.11 eV below the valence band maximum, showing the 7 pockets of the red valence band in (**a**). (**c**) Fermi surface calculated for a Fermi level at −0.11 eV below the valence band maximum, showing the 7 pockets of the blue valence band in (**a**).

Miao *et al*. have calculated the band structure of α-MgAgSb using the Perdew-Burke-Ernzerhof generalized gradient approximation (GGA) (GGA-PBE) exchange-correlation functional in the Vienna *ab-initio* simulation package (VASP), and predicted that α-MgAgSb is a semimetal^37^. Using the local density approximation (LDA) exchange-correlation potential as implemented in the VASP, Ying *et al*. found that α-MgAgSb is a semiconductor with an indirect band gap of 0.1 eV^38^. Because the GGA and LDA exchange correlation potentials always underestimate the band gap of crystals, we calculated the band structure of α-MgAgSb as implemented in WIEN2k with the Tran-Blaha modified Becke-Johnson (TB-mBJ) exchange correlation potential, as is shown in Fig. 4(a). Because the electron transport is closely related to the electronic states near the valence band maximum (VBM) and conduction band minimum (CBM), we only focused on the electronic states near the Fermi level. As shown in Fig. 4(a), α-MgAgSb is a semiconductor with a band gap of 0.32 eV, and the band structure is characterized by an indirect band gap, with the CBM near the Γ point and the VBM between Z and A. Sheng *et al*. also calculated the band structure by using the mBJ functional and the Heyd-Scuseria-Ernzerhof (HSE) approach as implemented in VASP^42^. They did not consider the *k*-path of Z-A, however, so they thought that the VBM was at X. As shown in Fig. 4(a), the VBM should be located at Z-A, and the maximum of the second valence band is located at M and X. The top of the valence band has a stronger dispersion than the bottom of the conduction band. The band dispersion relationship determines the effective mass, and the band mass of a single valley can be obtained by the following:

9 $${m}^{\ast }={\hslash }^{2}{[\frac{{{\rm{d}}}^{2}E(k)}{{\rm{d}}{k}}]}_{E(k)={E}_{F}}^{-{\rm{1}}}$$

where *k* is the wave vector, *E*_F_ is the Fermi energy, and *ħ* is the reduced Planck’s constant. According to Eq. (9), we know that the band effective mass at the top of the valence band is smaller than that at the bottom of the conduction band. Such large band dispersion of the valence band is conducive to the transmission of electrons. The small effective mass of top valence bands is helpful for increasing the electrical conductivity of *p*-type α-MgAgSb, although electrical conductivity is also determined by the carrier concentration.

As is well known, the maximum *ZT* of a material depends on the dimensionless thermoelectric quality factor $B\propto \mu {m}_{DOS}^{\ast 3/2}/{\kappa }_{L}$ ^48^, where *μ* is the mobility of the carrier and ${m}_{DOS}^{\ast }$ is the density-of-states (DOS) effective mass. The relationship between the density of states effective mass, the band degeneracy, *N*_*V*_, and the band effective mass, ${m}_{b}^{\ast }$, is given by: ${m}_{DOS}^{\ast }={N}_{V}^{2/3}{m}_{b}^{\ast }$. If acoustic phonon scattering dominates the carrier transport, then $\mu \propto 1/{m}_{b}^{\ast 3/2}{m}_{I}^{\ast }$ and $B\propto {N}_{V}/{m}_{I}^{\ast }{\kappa }_{L}$, where ${m}_{I}^{\ast }$ is the inertial mass. Thus, a large *N*_*V*_ is beneficial to a large ${m}_{DOS}^{\ast }$ without deterioration of *μ* ^6^. The band degeneracy *N*_*V*_ is based on the effective total number of independent carrier pockets or valleys in the Brillouin zone, including both orbital and symmetry related degeneracy. We adopted the strategy of increasing *N*_*V*_ for a high *ZT* as an example, as was well demonstrated for PbTe^6^. As a result of heavy hole doping and relatively light bands at the VBM, the Fermi level quickly moves down into the valence band, allowing a large population of holes to form in the secondary valence band. The calculations show that the secondary VBM is located at about −0.11 eV below the VBM. The Fermi surface calculations for a Fermi level −0.11 eV below the VBM of the red valence band and of the blue valence band are shown in Fig. 4(b) and (c), respectively. Figure 4(b) shows 8 half-pockets along Z-A, 4 quarter pockets at the M point, and 4 half-pockets at the X point so that the full number of valleys is 7. Figure 4(c) also shows that the full number of valleys is 7. Therefore, the iso-energy Fermi surface for an energy level at −0.11 eV has a high degeneracy with 14 isolated pockets. The large band degeneracy *N*_V_ may contribute to the high Seebeck coefficient at relatively high carrier concentrations. Based on above analysis, the large band dispersion of the valence band, together with the high band degeneracy with *N*_*V*_ = 14, may be the most significant feature that contributes to the good thermoelectric performance of *p*-type heavily doped α-MgAgSb.

**Figure 5:**
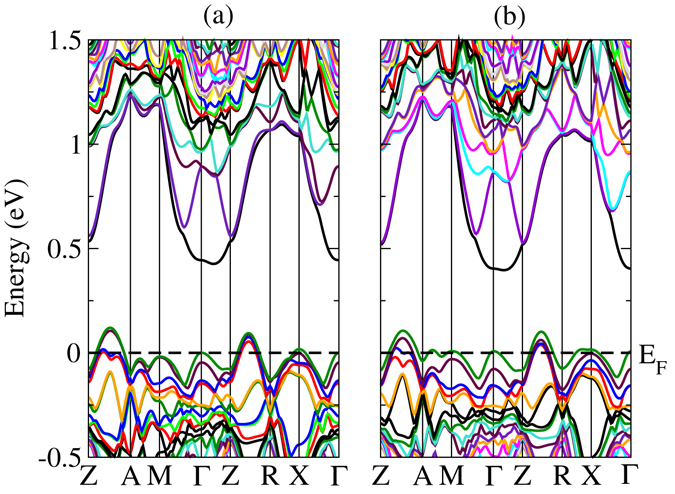
Calculated band structures of (**a**) Mg_48_Ag_47_Sb_48_ with Ag vacancy, and (**b**) Mg_47_Ag_49_Sb_48_ with Ag antisite. The top of the valence band is set to zero.

Figure 5(a) and (b) shows the band structures of Mg_48_Ag_47_Sb_48_ with an Ag vacancy and Mg_47_Ag_49_Sb_48_ with an Ag_Mg_ antisite, respectively. Both the Ag vacancy and the Ag antisite can break the symmetry of the supercell when introduced into the system. V_Ag_ and Ag_Mg_ show typical *p*-type doping behavior by shifting the Fermi level into the valence bands. The number of band valleys near the Fermi level increases. The large band degeneracy *N*_V_ and the heavy band effective mass can jointly contribute to the high Seebeck coefficient. Moreover, for *p*-type α-MgAgSb, the carrier concentration largely depends on the number of band valleys near the Fermi level. A high carrier concentration may help to increase the electrical conductivity. Therefore, the native defects V_Ag_ and Ag_Mg_ may play an important role in achieving a higher Seebeck coefficient and higher electrical conductivity, which will lead to a large *ZT* for α-MgAgSb. α-MgAgSb with the Ag_Mg_ defect has a larger number of band valleys near the Fermi level than with the V_Ag_ defect, which may lead to a larger *ZT* than with V_Ag_.

### Li-doped α-MgAgSb

Previous experimental work showed that Li doping can significantly increase the *ZT* value of α-MgAg_0.97_Sb_0.99_^39^. After Li doping, the carrier concentration increased from 2.3 × 10^19^ cm^−3^ to 1.4 × 10^20^ cm^−3^. The achieved average *ZT* was 1.1 from 300 to 548 K. The authors noted that Li was substituted onto the Mg sites^39^. It is valuable to explore how the chemical potential affects the doping sites in Li-doped α-MgAgSb. We calculated the formation energies of Li-doped α-MgAgSb as a function of chemical potential. For Li doping, the chemical potentials of impurities should satisfy other constraints to avoid the formation of impurity-related phases (such as Li source element, LiAg, Li_2_Sb, or Li_3_Sb):

10 $${\rm{\Delta }}{\mu }_{{Li}} < 0,$$

11 $${\rm{\Delta }}{\mu }_{Li}\,+\,{\rm{\Delta }}{\mu }_{Ag} < {\rm{\Delta }}{H}_{f}(LiAg),$$

12 $$2{\rm{\Delta }}{\mu }_{{Li}}+{\rm{\Delta }}{\mu }_{{Sb}} < {\rm{\Delta }}{H}_{f}({L}{{i}}_{2}{Sb}),$$

13 $$3{\rm{\Delta }}{\mu }_{{Li}}+{\rm{\Delta }}{\mu }_{{Sb}} < {\rm{\Delta }}{H}_{f}({L}{{i}}_{3}{Sb}).$$

Based on the representative chemical potential points, we can calculate the chemical potential of Li, and the values of the chemical potentials at points A and B are −0.8857 eV and −0.8163 eV for ${\rm{\Delta }}{\mu }_{{Li}}$, respectively. Then, the chemical potential is used for calculating the formation energy for Li-related defects. The impurities can either be at interstitial sites or substitute for Mg, Ag, or Sb. Therefore four different point defects, Li_Mg_, Li_Ag_, Li_Sb_, and Li_I_, have been included in our calculation. Because of the large formation energy for Li_Sb_, we only show the Li_Sb_ with zero charges.

**Figure 6:**
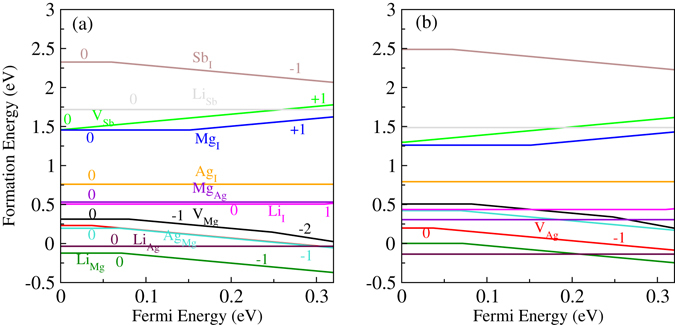
The calculated formation energies for Li doping and various native point defects in α-MgAgSb as a function of the Fermi level, with chemical potentials at (**a**) point A and (**b**) point B.

The calculated impurity formation energies of the doping systems are plotted in Fig. 6. As shown in Fig. 6, formation energies strongly depend on the chemical potentials. The thermodynamic transition level between ${{\rm{Li}}}_{{\rm{I}}}^{0}$ and ${{\rm{Li}}}_{{\rm{I}}}^{-1}$ is 0.01 eV below the CBM, indicating that Li_I_ is a shallow donor. At point A, substitutional Li on Mg sites has the lowest formation energy. At point B, substitutional Li on Ag sites has much lower formation energy than on Mg sites. Thus, at point A, Li doping can lead to good *p*-type conductivity, while at point B, Li doping cannot change the conductivity type of α-MgAgSb because of Li_Ag_ with zero charges. The formation energy for Li substitution on Mg sites at point A is smaller than for substituting Li atoms on Ag sites or Sb sites, implying that the Li atoms prefer to occupy the Mg sites rather than the Ag or Sb sites at point A. On the other hand, at point B (more Mg-rich conditions), Li substitution on Ag sites of α-MgAgSb is the most stable structure. Therefore, we can substitute Li on Mg sites or Ag sites in α-MgAgSb by controlling the chemical potential of Li under different conditions.

**Figure 7:**
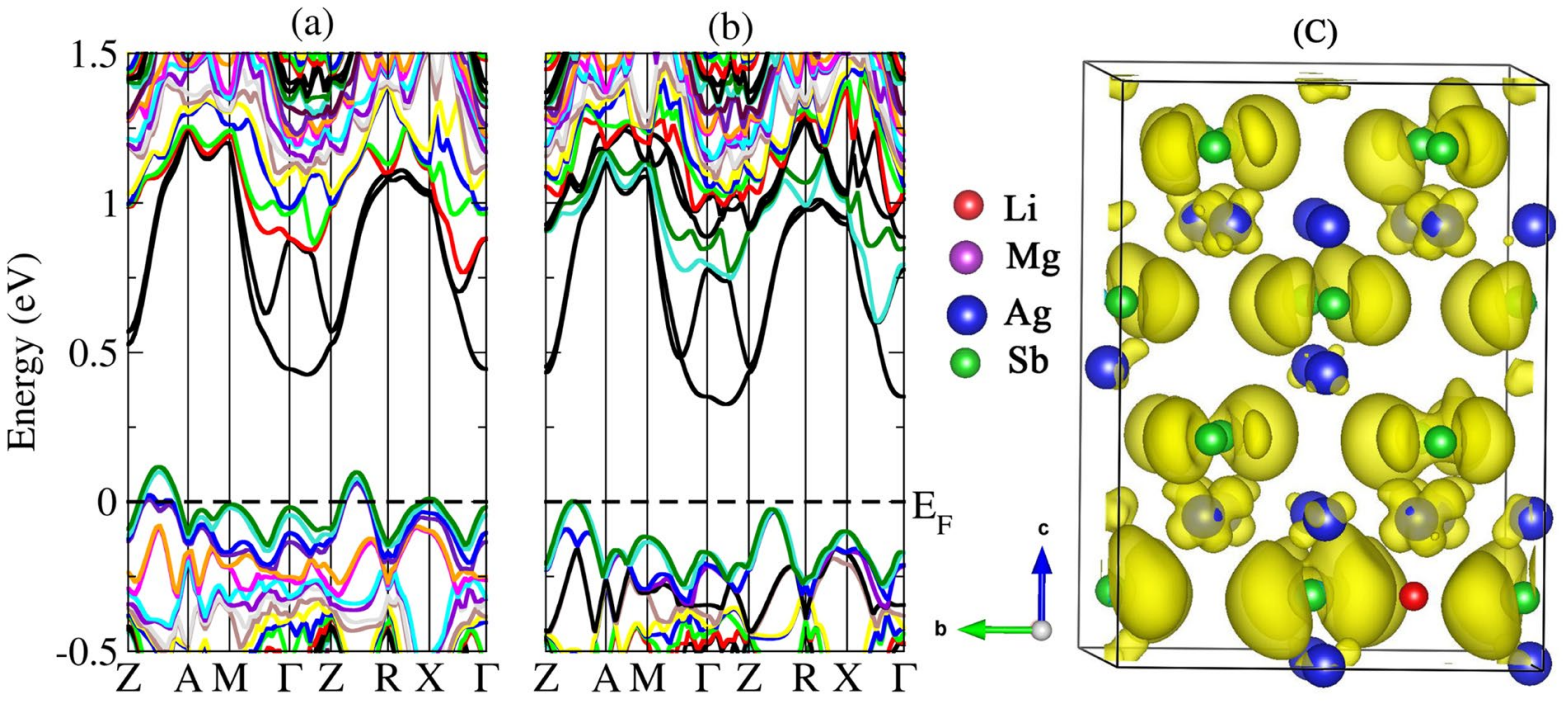
Calculated band structures of (**a**) Mg_47_LiAg_48_Sb_48_ and (**b**) Mg_48_Ag_47_LiSb_48_. The dashed line denotes the Fermi level. (**c**) Calculated band-decomposed charge density of Mg_0.98_Li_0.02_AgSb for valence bands near the Fermi level at point Γ, with the isosurface value of 0.0009 eÅ^−3^.

We also investigated the electronic structure and the transport properties for α-MgAgSb with Li doping on Mg sites and Ag sites at a doping level of 0.02. The calculated band structures of Mg_47_LiAg_48_Sb_48_ and Mg_48_Ag_47_LiSb_48_ are shown in Fig. 7(a) and (b), respectively. As shown in Fig. 7(a), the most obvious change from substituting Li on Mg sites is the appearance of large valley degeneracy in the valence bands near the Fermi level, and the band gap of Li-doped α-MgAgSb is 0.32 eV. The good thermoelectric properties of a thermoelectric material depend on the weighted carrier mobility, $\mu {({m}_{DOS}^{\ast }/{m}_{e})}^{3/2}$; the density of states effective mass is defined by ${m}_{{DOS}}^{\ast }={N}_{V}^{2/3}{m}_{b}^{\ast }$. Note that the carrier mobility is strongly affected by the band mass of a single valley: $\mu \,\propto \,1/{m}_{b}^{\ast 5/2}$ ^49^. Therefore, increasing the band mass should be detrimental to the carrier mobility. Multiple degenerate valleys may produce a large ${m}_{DOS}^{\ast }$ without explicitly reducing the carrier mobility. In that case, a large valley degeneracy is helpful for the thermoelectric material^6^. As can be seen in Fig. 7(a), the Fermi level moves down into the valence band by 0.11 eV because of Li doping on Mg sites in α-MgAgSb, and the energy of the Γ point becomes higher towards the Fermi level so that the number of band valleys near the Fermi level increases.

To explain the reason why the Γ point becomes higher and moves toward the Fermi level, we calculated the partial charge densities near the Fermi level at the Γ point using VASP, as shown in Fig. 7(c). Because there is little charge density distribution around the Mg atoms, we do not display the Mg atoms. From the shape of the charge density, we can see that the states near the Fermi level at the Γ point mainly come from the Sb *p* orbitals. The large band degeneracy *N*_V_ and heavy band effective mass can jointly contribute to the high Seebeck coefficient. Moreover, for *p*-type α-MgAgSb, the carrier concentration largely depends on the number of band valleys near the Fermi level, which is mainly due to the fact that more carriers can be activated across the band gap. High carrier concentration may help to increase the electrical conductivity. Therefore, Li doping may play an important role in achieving a higher Seebeck coefficient and electrical conductivity, which will lead to a large *ZT* for Mg_47_LiAg_48_Sb_48_. Recently, Liu *et al*. used Li doping to increase the carrier concentration of MgAg_0.97_Sb_0.99_, thus decreasing the electrical resistivity, and a high average *ZT* ^39^ of 1.1 from 300 K to 548 K was achieved.

The calculated band structure of Mg_48_Ag_47_LiSb_48_ is shown in Fig. 7(b). As can be seen in Fig. 7(b), the number of band valleys near the Fermi level increases because the energy along Z-R becomes higher towards the Fermi level. Multiple degenerate valleys may produce a large ${m}_{DOS}^{\ast }$, and a large ${m}_{DOS}^{\ast }$ may lead to a large Seebeck coefficient.

### Elastic and thermal properties

Ying *et al*. found that the appearance of three-centered Mg-Ag-Sb bonds in α-MgAgSb results in low intrinsic lattice thermal conductivity^50^. To investigate the elastic properties of α-MgAgSb with intrinsic defects and Li doping, the stress-strain method was used to calculate the elastic constants and other elastic properties^51^. A small finite strain is applied on the optimized structure, and then the atomic positions are optimized. The elastic constants are obtained from the stress of the strained structure. The calculated elastic constants of MgAgSb and Mg_0.98_Li_0.02_AgSb are listed in Table 1. It is clearly seen that all the studied compounds satisfy the mechanical stability criteria^52^, indicating that they are elastically stable. On the other hand, the positive eigenvalues of the elastic constant matrix for each compound further prove that they are elastically stable. From the calculated elastic constants *C*_ij_, the polycrystalline bulk modulus *B* and shear modulus *G* were estimated using the Voigt-Reuss-Hill approximation^53^. A high (low) *B*/*G* ratio of a material indicates that it is ductile (brittle), and the critical value is about 1.75^54^. The calculated *B*/*G* ratios for MgAgSb and Mg_0.98_Li_0.02_AgSb are larger than the critical value (1.75), indicating that they are all ductile materials.

**Table 1:** Calculated elastic constants (*C*_ij_ in GPa), theoretical density (*ρ* in g/cm^3^), bulk modulus (*B* in GPa), shear modulus (*G* in GPa), shear sound velocity (*v*_*s*_ in km/s), longitudinal sound velocity (*v*_*l*_ in km/s), Debye temperature (*Θ*_D_ in K), and minimum lattice thermal conductivity (*κ*_min_ in W/mK) of α-MgAgSb, V_Ag_ (Mg_48_Ag_47_Sb_48_), Ag_Mg_ (Mg_47_Ag_49_Sb_48_), Li_Mg_ (Mg_48_LiAg_48_Sb_48_), and Li_Ag_ (Mg_48_Ag_47_LiSb_48_).

	C_11_	C_12_	C_13_	C_33_	C_44_	C_66_	ρ	B	G	ν_s_	ν_l_	Θ_D_	κ_min_
MgAgSb	89	34	47	70	30	18	6.31	56.2	26.2	2.04	3.80	241	0.55
V_Ag_	87	32	49	70	27	14	6.08	56.5	23.6	1.97	3.80	231	0.53
Ag_Mg_	90	36	48	72	28	17	6.18	58.2	25.6	2.03	3.86	239	0.54
Li_Mg_	87	36	48	68	27	16	6.13	56.4	23.9	1.97	3.79	232	0.53
Li_Ag_	93	35	49	69	29	16	6.09	57.9	25.3	2.04	3.88	239	0.54

Thermal conductivity of a material includes both electronic and lattice thermal conductivity. The electronic contribution to the thermal conductivity is described by the Wiedemann-Franz relation, ${\kappa }_{e}=LT\sigma$, where L is the Lorenz number. Above the Debye temperature, the lattice thermal conductivity is generally limited by Umklapp scattering, which leads to ${\kappa }_{l}\propto 1/{T}$. This 1/*T* decay can only continue, however, until the minimum lattice thermal conductivity (*κ*_*min*_) is reached, as defined by Cahill^55, 56^. At high temperature (*T* > *Θ*_*D*_), *κ*_*min*_ can be approximated by the following formula:

14 $${\kappa }_{{\min }}=\frac{1}{2}[{(\frac{\pi }{6})}^{\frac{1}{3}}]{k}_{B}({V}^{-\frac{2}{3}})({\rm{2}}{\nu }_{s}+{\nu }_{l}),$$

where *V* is the average volume per atom, and *v*_*s*_ and *v*_*l*_ are the shear and longitudinal sound velocities, respectively. As a fundamental parameter, the Debye temperature is connected with many physical properties of solids, such as the specific heat, melting point, and elastic constant. At low temperatures, the vibrational excitations arise solely from acoustic vibrations. One of the methods used to calculate the Debye temperature is based on the elastic constant data. The Debye temperature is given by:

15 $${\Theta }_{D}=\frac{h}{{k}_{B}}[\frac{{\rm{3}}n}{4{\rm{\pi }}}(\frac{{N}_{A}\rho }{M})]{\nu }_{m},$$

where *k*_*B*_, *h*, *N*_*A*_, *ρ*, *M*, and *n* are the Boltzmann constant, Planck’s constant, Avogadro’s number, density, molecular weight of the solid, and number of atoms in the molecule, respectively. The average wave velocity *v*_*m*_ in polycrystalline materials is approximately given by^57^

16 $${\nu }_{m}={[\frac{1}{3}(\frac{2}{{\nu }_{s}^{3}}+\frac{1}{{\nu }_{l}^{3}})]}^{-1/3}.$$

*v*_*s*_ and *v*_*l*_ can be obtained using the polycrystalline shear modulus *G* and the bulk modulus *B* from Navier’s equation as follows^58^:

17 $${\nu }_{s}=\sqrt{\frac{G}{\rho }}\,{\rm{and}}\,{\nu }_{l}\,=\sqrt{\frac{B+\frac{4}{3}G}{\rho }},\,$$

*B* and *G* can estimate using the Voigt-Reuss-Hill approximation from the calculated elastic constant data, which were obtained by the stress-strain method^53^. The calculated elastic constants and the minimum lattice thermal conductivity are listed in Table 1.

For α-MgAgSb, V_Ag_ (Mg_48_Ag_47_Sb_48_), Ag_Mg_ (Mg_47_Ag_49_Sb_48_), Li_Mg_ (Mg_48_LiAg_48_Sb_48_), and Li_Ag_ (Mg_48_Ag_47_LiSb_48_), the calculated *κ*_*min*_ values are 0.55 W/mK, 0.53 W/mK, 0.54 W/mK, 0.53 W/mK, and 0.54 W/mK, respectively. As shown in Eq. 14, the minimum lattice thermal conductivity is strongly affected by the shear sound velocity. Table 1 shows that the V_Ag_ and Li_Mg_ defects induce an obviously decreasing shear modulus in α-MgAgSb, which indicates that V_Ag_ and Li_Mg_ defects weaken the resistance against shear deformation of α-MgAgSb. Thus, the shear sound velocity decreases due to Ag vacancy and Li_Mg_ doping. Consequently, the minimum lattice thermal conductivity values are reduced due to Ag vacancy and Li_Mg_ doping. For Ag_Mg_ and Li_Ag_ defects, the decrease in the shear modulus is not so large compared with V_Ag_ and Li_Mg_ defects. Thus, the change in the minimum lattice thermal conductivity due to Ag_Mg_ and Li_Ag_ defects is smaller than that due to V_Ag_ and Li_Mg_ defects.

### Electrical transport properties

A material with a large *ZT* needs to have a large *S* (found in low carrier concentration semiconductors or insulators) and a large *σ* (found in high carrier concentration metals). The carrier concentration dependence of the Seebeck coefficient and the electrical conductivity are shown in Eqs (18) and (19), respectively^2^. In these equations, *T* is the temperature, and *μ* is the charge carrier mobility.

18 $$S\,=\frac{{\rm{8}}\pi {k}_{B}^{2}}{{\rm{3}}e{h}^{2}}{m}_{DOS}^{\ast }T{(\frac{\pi }{{\rm{3}}n})}^{\frac{2}{3}},$$

19 $$\sigma \,=ne\mu $$

**Figure 8:**
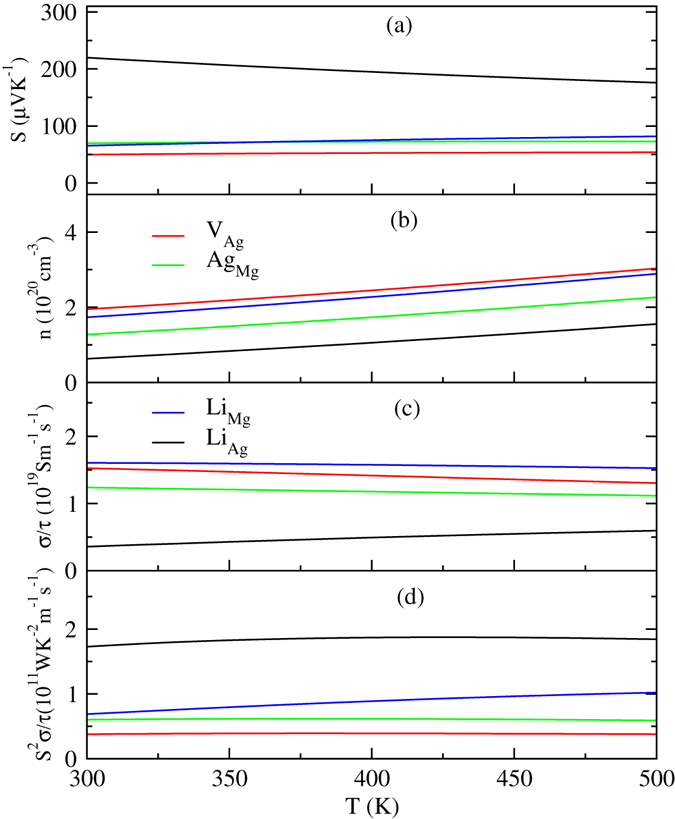
Calculated thermoelectric properties of various defects in α-MgAgSb as a function of temperature.

Equation (18) suggests that the Seebeck coefficient is proportional to the temperature and ${m}_{DOS}^{\ast }$, yet is inversely related to the carrier concentration. The electrical conductivity is proportional to the carrier concentration and inversely proportional to the effective mass. We calculated the Seebeck coefficient, *S*, the carrier concentration, *n*, the electrical conductivity relative to relaxation, σ/τ, the thermopower relative to relaxation, and the figure of merit, S^2^σ/τ, as a function of temperature, as shown in Fig. 8.

As can be seen in Fig. 8(a), the Seebeck coefficients of V_Ag_ (Mg_48_Ag_47_Sb_48_), Ag_Mg_ (Mg_47_Ag_49_Sb_48_), Li_Mg_ (Mg_48_LiAg_48_Sb_48_), and Li_Ag_ (Mg_48_Ag_47_LiSb_48_) are all positive over the entire studied temperature range, indicating *p*-type transport for the four types of defects. The *n* and *σ*/*τ*, of Ag_Mg_ are lower than those of V_Ag_, while Ag_Mg_ has the larger *S* ^*2*^ *σ*/*τ* owing to its large *S*. Liu *et al*. reported that Ag vacancy can be rationally engineered by controlling the hot pressing temperature, and a high peak *ZT* of ~1.4 and an average *ZT* of ~1.1 can be achieved^30^. α-MgAgSb containing Ag_Mg_ point defects may have higher *ZT* than with Ag vacancy because α-MgAgSb with Ag_Mg_ has a larger ${S}^{2}\sigma /\tau$ than with Ag vacancy. The *S* of Li_Ag_-doped α-MgAgSb is larger than for Li_Mg_-doped α-MgAgSb. Although Li_Ag_-doped α-MgAgSb has the lowest *n* and *σ*/*τ*, the *S* ^*2*^ *σ*/*τ* of Li_Ag_-doped α-MgAgSb is larger than that with Li_Mg_ defects, due to the large *S*, as shown in Fig. 8(a). Liu *et al*. found that the average *ZT* can reach as high as 1.1 from 300 K to 548 K when there is Li doping on Mg sites of MgAg_0.97_Sb_0.99_^39^. Thus, under more Mg-rich conditions, Li_Ag_ doping may lead to a larger *ZT* than for Li substitution on Mg sites. Thus, good thermoelectric performance as a result of the antisite defect Ag_Mg_ and as a result of Li substitution on Ag sites in α-MgAgSb can be predicted.

## Conclusions

In this work, we investigated the defect formation energies, the electronic structure, and the thermoelectric performance of the host α-MgAgSb and the effects of substitutional Li doping of α-MgAgSb, by using density functional theory combined with semiclassical Boltzmann theory. We found that the formation energies strongly depend on the chemical potentials. Ag vacancy and Ag-Mg antisite defects are the dominant defects, acting as the shallow acceptors that determine the *p*-type conduction of experimentally synthesized α-MgAgSb. Moreover, for α-MgAgSb, the Ag_Mg_ antisite defect may induce a higher *ZT* than Ag vacancy, due to the more numerous band valleys near the Fermi level than with Ag_Mg_ in α-MgAgSb. α-MgAgSb has a secondary valence band with 14 carrier pockets, which indicates that heavily *p*-type doping may lead to a high thermoelectric performance in α-MgAgSb. For Li-doped α-MgAgSb, Li doping on Ag sites has a lower formation energy than on Mg sites under more Mg-rich conditions, and Li_Ag_ may lead to a larger *ZT* than for Li doping on Mg sites. Thus, engineering atomic scale defects is an effective strategy for enhancing the thermoelectric properties of α-MgAgSb, and the achieved high *ZT* demonstrates that Ag_Mg_ antisite defects and the substitution of Li on Ag sites in α-MgAgSb could lead to materials with good potential for future application in the thermoelectric area.

## Computational Details

The electronic structure of α-MgAgSb was investigated using the full-potential linearized augmented plane wave method^59^, as implemented in WIEN2k^60–62^. The Tran and Blaha modified semi-local Becke–Johnson exchange correlation potential (TB-mBJ)^63^ was used, which is known to give much more accurate band gaps than the standard Engel–Vosko generalized-gradient approximation (EV-GGA)^64^. The muffin-tin radii were chosen to be 2.5 a.u. for Mg, Ag, and Sb. The cut-off parameter *R*_mt_ × *K*_max_ = 9 (where *K*_max_ is the magnitude of the largest *k* vector) was used, and the self-consistent calculations were performed with 2000 k-points in the irreducible Brillouin zone; the total energy was made to converge to within 1 mRy. The electrical transport properties were then calculated by using semiclassical Boltzmann theory^65, 66^ within the constant scattering time approximation, as implemented in the Boltzmann Transport Properties (Boltz-TraP) code^67^. This approximation has been used to calculate the transport coefficients of some known thermoelectric materials and very good agreement with experimental results was achieved^68, 69^.

We simulated various defects in α-MgAgSb, along with Li doping, using a supercell that contained 144 atoms. We considered three intrinsic point defects, vacancy, interstitial, and antisite. Because of their large formation energies, cation/anion antisites, such as Mg or Ag on the Sb site and Sb on the Mg or Ag sites, are not discussed in this study. The intrinsic defects considered in this study include V_Ag_ (Ag vacancy), V_Mg_ (Mg vacancy), V_Sb_ (Sb vacancy), Mg_I_ (Mg interstitial), Ag_I_ (Ag interstitial), Sb_I_ (Sb interstitial), Ag_Mg_ (Ag on Mg site), and Mg_Ag_ (Mg on Ag site). In the case of Li doping, we simulated interstitial doping (Li_I_) and substitutional doping, including Li_Mg_ (Li doping on Mg site), Li_Ag_ (Li doping on Ag site), and Li_Sb_ (Li doping on Sb site).

**Figure 9:**
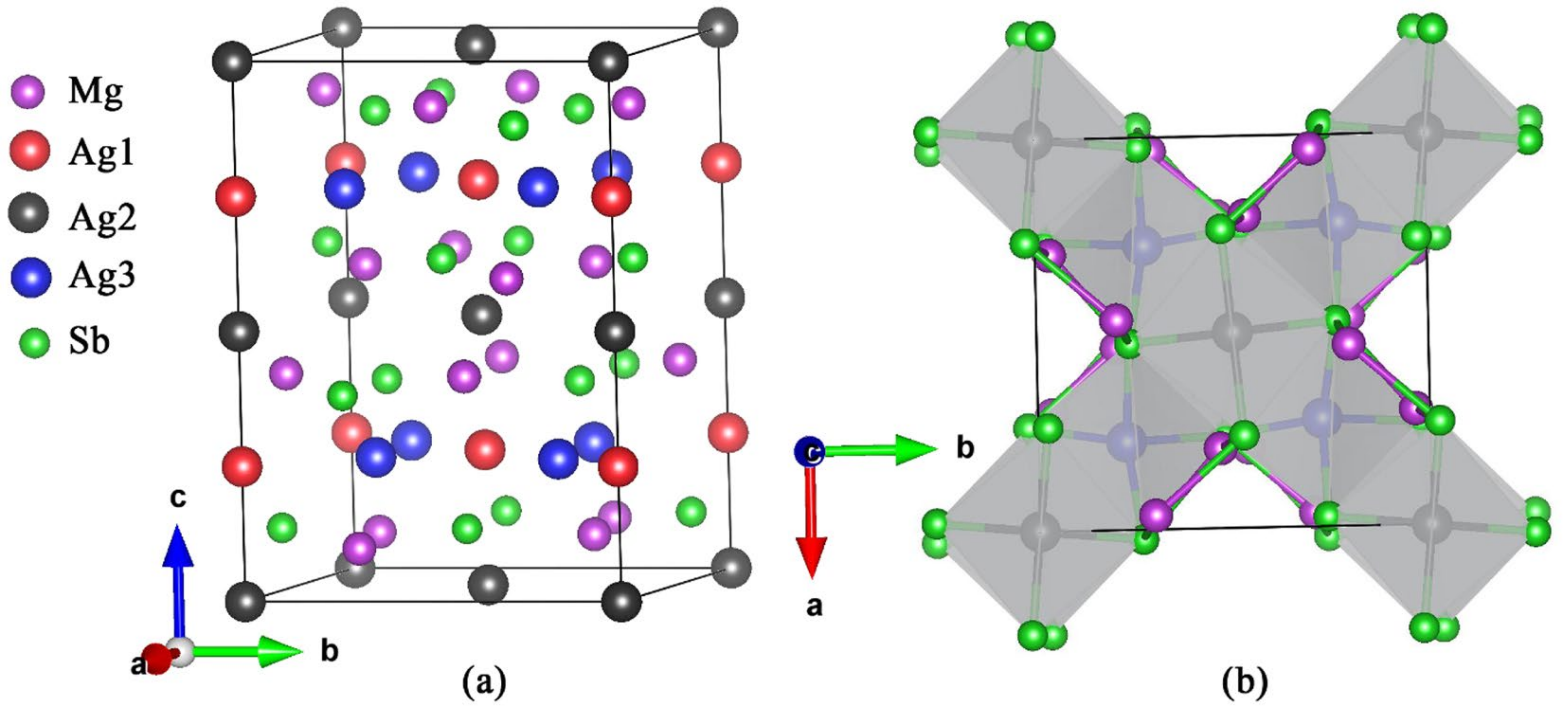
Crystal structure of α-MgAgSb, looking along the (**a**) [110] and (**b**) [001] directions. Mg, Ag1, Ag2, Ag3, and Sb represent the five crystallographically unique sites of α-MgAgSb.

As shown in Fig. 9, there are 48 atoms in each unit cell of α-MgAgSb, which contains five crystallographically unique atomic sites: one Mg, three Ag, and one Sb. The structural parameters of α-MgAgSb are shown in Table 2. α-MgAgSb consists of a distorted Mg-Sb rock-salt lattice, rotated by 45° about the *c* axis, with half of the Mg-Sb pseudocubes filled with Ag, although the pseudocubes where silver atoms are located are quite different from those in half-Heusler compounds^70^. Such a complex lattice structure may lead to a relatively small thermal conductivity.

**Table 2:** Lattice constants and atomic coordinates of α-MgAgSb. Ag1, Ag2, and Ag3 represent three crystallographically unique Ag sites.

Lattice parameter	Atomic type	x	y	z
Crystal system: Tetragonal	Mg	0.97357	0.27610	0.11456
Space group: ${\rm{I}}\bar{4}{\rm{c2}}$ (NO. 120)	Ag1	0.0000	0.00000	0.25000
*a* = 9.2816 Å	Ag2	0.0000	0.0000	0.00000
*c* = 12.7481 Å	Ag3	0.22158	0.22158	0.25000
	Sb	0.23158	0.47522	0.11586

We also studied the electronic structure and thermoelectric properties of V_Ag_, Ag_Mg_, Li_Mg_, and Li_Ag_ using the supercell (144 atoms in MgAgSb supercell), corresponding to a doping level of 2% for α-MgAgSb. We also fixed the lattice constants, only optimizing the internal coordinates. The electronic structures of Mg_48_Ag_47_Sb_48_, Mg_47_Ag_49_Sb_48_, Mg_47_LiAg_48_Sb_48_, and Mg_48_Ag_47_LiSb_48_ were calculated with WIEN2k. Other parameters were in accordance with the calculations for α-MgAgSb.
